# Agnosia for accents in primary progressive aphasia^☆^

**DOI:** 10.1016/j.neuropsychologia.2013.05.013

**Published:** 2013-08

**Authors:** Phillip D. Fletcher, Laura E. Downey, Jennifer L. Agustus, Julia C. Hailstone, Marina H. Tyndall, Alberto Cifelli, Jonathan M. Schott, Elizabeth K. Warrington, Jason D. Warren

**Affiliations:** Dementia Research Centre, UCL Institute of Neurology, University College London, London, United Kingdom

**Keywords:** Primary progressive aphasia, Dementia, Accent processing, Phonagnosia, Prosopagnosia

## Abstract

As an example of complex auditory signal processing, the analysis of accented speech is potentially vulnerable in the progressive aphasias. However, the brain basis of accent processing and the effects of neurodegenerative disease on this processing are not well understood. Here we undertook a detailed neuropsychological study of a patient, AA with progressive nonfluent aphasia, in whom agnosia for accents was a prominent clinical feature. We designed a battery to assess AA's ability to process accents in relation to other complex auditory signals. AA's performance was compared with a cohort of 12 healthy age and gender matched control participants and with a second patient, PA, who had semantic dementia with phonagnosia and prosopagnosia but no reported difficulties with accent processing. Relative to healthy controls, the patients showed distinct profiles of accent agnosia. AA showed markedly impaired ability to distinguish change in an individual's accent despite being able to discriminate phonemes and voices (apperceptive accent agnosia); and in addition, a severe deficit of accent identification. In contrast, PA was able to perceive changes in accents, phonemes and voices normally, but showed a relatively mild deficit of accent identification (associative accent agnosia). Both patients showed deficits of voice and environmental sound identification, however PA showed an additional deficit of face identification whereas AA was able to identify (though not name) faces normally. These profiles suggest that AA has conjoint (or interacting) deficits involving both apperceptive and semantic processing of accents, while PA has a primary semantic (associative) deficit affecting accents along with other kinds of auditory objects and extending beyond the auditory modality. Brain MRI revealed left peri-Sylvian atrophy in case AA and relatively focal asymmetric (predominantly right sided) temporal lobe atrophy in case PA. These cases provide further evidence for the fractionation of brain mechanisms for complex sound analysis, and for the stratification of progressive aphasia syndromes according to the signature of nonverbal auditory deficits they produce.

## Highlights

• Deficits of accent processing can be a presenting feature of primary progressive aphasia.
• Accent agnosia can dissociate from a generalised auditory agnosia.
• Progressive aphasia syndromes show different profiles of accent processing deficits.

## 1 Introduction

The progressive aphasias (PPA) are a diverse group of neurodegenerative syndromes with characteristic clinico-anatomical signatures and heterogeneous histopathology (Mesulam, 1982; Gorno-Tempini et al., 2008, 2011). Three canonical PPA syndromes are recognised (Gorno-Tempini et al., 2011): progressive nonfluent aphasia (PNFA), characterised by impaired speech production and agrammatism associated with predominant left peri-Sylvian atrophy; semantic dementia (SD), characterised by impaired single word comprehension and loss of vocabulary, associated with asymmetric, selective anterior temporal lobe atrophy; and logopenic aphasia (LPA), characterised by prolonged word-finding pauses and impaired auditory verbal working memory, associated with predominant left temporo-parietal atrophy. By definition, PPA syndromes are primarily defined by language deficits; however, nonverbal deficits are increasingly recognised and are likely to be integral to the pathophysiology of PPA, reflecting a profile of brain network disintegration in these diseases. Examples of such non-linguistic impairments include the breakdown of multi-modal object and conceptual knowledge in SD (Bozeat, Lambon Ralph, Patterson, Garrard, & Hodges, 2000; Goll et al., 2010a; Goll, Crutch, & Warren, 2010b; Goll, Ridgway, Crutch, Theunissen, & Warren, 2012; Hailstone, Crutch, Vestergaard, Patterson, & Warren, 2010; Luzzi et al., 2007; Omar, Hailstone, Warren, Crutch, & Warren, 2010; Piwnica-Worms, Omar, Hailstone, & Warren, 2010; Josephs, 2008; Fletcher & Warren, 2011) and deficits of nonverbal sound processing across the PPA spectrum (Hailstone et al., 2010, 2011, 2012; Goll et al., 2010a,2011; Rohrer, Sauter, Scott, Rossor, & Warren 2012). With respect to nonverbal sounds, deficits in PPA syndromes span a hierarchy of early perceptual, apperceptive and semantic processing stages, analogous to the processing hierarchy established for visual objects (Warrington & Taylor, 1973; Warrington, 1982; Warrington & Taylor, 1978; Riddoch & Humphreys, 1987; Griffiths & Warren, 2002, 2004; Goll et al., 2010a). Particular PPA syndromes are associated with distinctive profiles of nonverbal auditory deficits: Whereas auditory apperceptive and semantic impairments have been demonstrated in both SD and PNFA, additional early auditory perceptual impairments occur in PNFA and more widespread auditory deficits have been documented in LPA (Goll et al., 2010a, 2011).

The processing of accents is potentially of particular relevance to understanding the PPA syndromes (Hailstone et al., 2012). Accent is a meta-linguistic feature of spoken utterances that conveys information about the speaker's geographical or socio-cultural background: accent is therefore potentially a rich source of nonverbal semantic information about speakers. In addition, accent modifies the acoustic properties of spoken phonemes, interacting with individual vocal characteristics and prosody (Boula de Mareuil & Vieru-Dimulescu, 2006; Clopper & Pisoni, 2004; Howell, Barry, & Vinson, 2006); if spoken phonemes are regarded as auditory objects (Griffiths & Warren, 2004), then a phoneme spoken in a non-native accent could be considered as a non-canonical ‘view’ of the phoneme for a particular listener, and should therefore engage auditory apperceptive processing. Both recognition of non-native accents and comprehension of words spoken with less familiar accents have been shown to be impaired in patients with PNFA, in keeping with conjoint semantic and apperceptive deficits of accent processing in this PPA syndrome (Hailstone et al., 2012). However, limited information is currently available concerning the brain basis of accent processing and the impact of disease on this processing. In particular, no detailed and systematic comparison of the processing of accent in relation to other kinds of complex auditory signals has previously been undertaken in PPA.

Here we describe a detailed analysis of the processing of accent in a patient, AA, with PNFA. Difficulties with accent recognition and comprehension were early and prominent features of AA's clinical syndrome. AA's performance on apperceptive and semantic analysis of accents, voices, speech and environmental sounds was assessed using a novel neuropsychological battery and compared with the performance of healthy control participants and another patient, PA, with a syndrome of SD characterised by progressive anomia, prosopagnosia and phonagnosia, but no reported difficulties with accent processing.

## 2 Methods

### 2.1 Participant details

Demographic data for all participants are summarised in Table 1.

#### 2.1.1 Patient AA

**Fig. 1:**
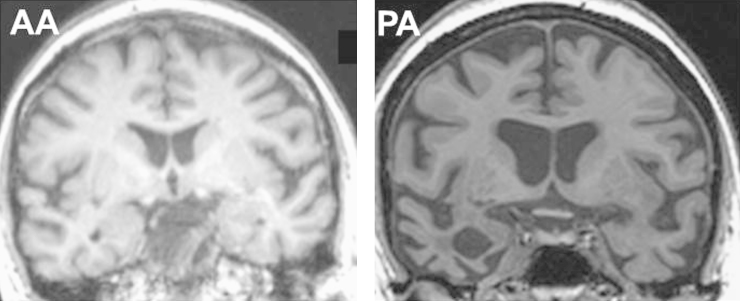
Representative coronal T1-weighted MR images from the patients (left hemisphere shown projected on the right side in each case): AA (left), showing asymmetric predominantly left-sided peri-Sylvian atrophy; and PA (right), showing asymmetric, predominantly right-sided anterior and mesial temporal lobe atrophy.

This 67 year old right handed retired teaching assistant, who had lived in the London area for the whole of her life, presented with a two year history of progressive word finding difficulty and hesitant, effortful speech. In addition, she had noticed prominent difficulty identifying a speaker's accent and in understanding non-native accents. For example, when watching a film or television programme she was unable to follow the conversation of actors speaking in foreign accents or to identify their accents. In the last year she had also experienced some difficulty recognising individual voices. This was particularly evident when using the telephone, though remained relatively mild in relation to her difficulties with accents. On examination her speech was nonfluent and agrammatic, with speech apraxia and frequent phonetic errors. She exhibited prominent orofacial apraxia; the general neurological examination was normal. When asked to identify the examiner's accent (Australian) she reported that she had not realised that this was non-native but when pushed, suggested that he might be ‘Northern’. AA fulfilled current clinical diagnostic criteria for the nonfluent-agrammatic variant of PPA, here designated PNFA (Gorno-Tempini et al., 2011). Brain MRI showed predominantly left-sided peri-Sylvian atrophy (Fig. 1).

#### 2.1.2 Patient PA

This 71 year old right handed retired medical secretary, who had lived in the South East region of England for the whole of her life, presented with a seven year history of progressive difficulty recognising people. When first assessed, she had difficulty recognising close relatives and friends. In addition, for the past two years she had developed difficulty recognising voices over the telephone and had begun to notice problems recalling the names of things. She had recently developed an obsessional interest in puzzles and crossword books. Family members also reported that she was less empathic. On examination her speech was garrulous and circumlocutory with anomia. The general neurological examination was unremarkable. PA was diagnosed clinically with a semantic dementia syndrome led by progressive prosopagnosia. Brain MRI showed marked bilateral anterior temporal lobe atrophy, more severe on the right (Fig. 1).

#### 2.1.3 Healthy control participants

Twelve healthy age and gender matched individuals (mean age 66 years, range 57–71 years) participated. All were native English speakers. Eleven had grown up in the South East of England and had lived in the London area for the majority of their lives; one participant had originally grown up in New York but had lived in London for the last forty years. No participant had a history of neurological or psychiatric illness. The healthy control group had, on average, higher educational attainment than the patients (see Table 1): The patients had 10 and 11 years of education (corresponding to finishing school aged 15 or 16, prior to O-Levels/G.C.S.E.s) whereas the control group had on average 16 years of education(corresponding to Degree level education).

All participants were recruited via the Cognitive Disorders Clinic at the National Hospital for Neurology and Neurosurgery. The study was approved by the local institutional research ethics committee and all participants gave informed consent in accord with the principles of the Declaration of Helsinki.

### 2.2 General neuropsychological assessment

A comprehensive assessment of general neuropsychological functions covering language, executive functions, working memory and posterior cortical cognitive domains was undertaken in all participants. Details of the neuropsychological tests administered are summarised in Table 1.

### 2.3 Experimental behavioural assessment

#### 2.3.1 Structure of the battery and general procedure

Apperceptive processing of accents (perception of a change in accent) was assessed in relation to perception of change in two other major categories of vocal auditory objects: phonemes and individual voice identity. Semantic (associative) processing of accents (accent identification) was assessed in relation to the identification of two other major categories of sound objects, individual voices and environmental sounds; and also in relation to the identification of faces, a key personal attribute in the visual modality.

Sound stimuli were recorded as digital wavefiles on a notebook computer, edited where required using Goldwave^®^ and presented under Matlab7.0^®^ via speakers at a comfortable listening level (at least 70 dB). Trials within each subtest were presented in randomised order. Participant responses were recorded for off-line analysis. Practice trials were administered before each subtest to ensure the participant understood the task. No feedback was given about performance during the test and no time limit was imposed.

#### 2.3.2 Apperceptive processing

##### 2.3.2.1 Perception of change in accent

In general, accent can only be varied by concomitantly varying speaker identity: here, we set out to separate processing associated with change in accent and change in speaker. We capitalised on the ability of a professional dialect coach (MT) to create stimuli in which accent was varied independently of vocal identity. MT recorded the same series of English words (listed in Supplementary Table S1 on-line) spoken under standard Southern English and under two non-native accents; East Coast American and Australian. Words were between one and four syllables long. Individual words were concatenated into sequences of four words in which accent either remained the same (standard Southern English) or changed mid-way through the sequence (to East Coast American or Australian). Examples of the stimuli are provided in Supplementary Material on-line. Thirty trials were presented, comprising 10 trials with constant accent and 20 with changing accent (11 American, 9 Australian). Each word was used at least once in both conditions. The task on each trial was to determine whether the accent altered or remained the same.

##### 2.3.2.2 Perception of change in phoneme

Word pairs derived from the Psycholinguistic Assessment of Language Processing in Aphasia (PALPA)-3 minimal pairs discrimination subtest (Kay, Lesser & Coltheart, 1992); listed in Supplementary Table S2 on-line) were presented in spoken form by one of the authors (PF) under a standard southern English accent. Thirty-six trials were delivered, half of these comprising word pairs based on the same word and half comprising word pairs based on different but phonetically similar words (e.g., ‘leaf’—‘leave’). The task on each trial was to determine whether the word altered or remained the same.

##### 2.3.2.3 Perception of change in speaker

This test has been described previously (Hailstone et al., 2010). Six females aged 21 to 31 each with a standard southern English accent were recorded speaking a short sequence of highly familiar words (‘Monday, Tuesday, Wednesday, Thursday’). These sequences were edited to fix word duration, presentation rate and loudness between speakers, and re-concatenated to create experimental trials in which the speaker either remained the same or changed mid-way through the sequence. 28 trials were delivered, half of these comprising word sequences based on the same speaker and half comprising word sequences based on different speakers. The task on each trial was to determine whether the speaker altered or remained the same.

#### 2.3.3 Semantic processing

##### 2.3.3.1 Accent identification

Identification of accents was assessed on spoken sentences derived from a short passage (Honorof, McCullough and Somerville, 2000 (Supplementary Table S3 on-line)) that was read aloud by the same professional dialect coach (MT) in five different accents: standard Southern English, Australian, East Coast American, South African and Chinese. The same six sentences were presented for each accent. The task on each sentence trial was to identify the accent from one of the five alternative accent names presented in written form and also spoken aloud by the examiner. Both patients were first asked to read the written accent name and provide some associative information for that country such as famous exports or animals to assess geographical knowledge. Neither patient exhibited a frank deficit on this task and both were able to provide sufficient information to complete the task.

##### 2.3.3.2 Voice identification

Identification of voices was assessed using a previously described test based on a set of 24 famous voices well known to older British individuals (Hailstone et al., 2010; listed in Supplementary Table S4 on-line). Vocal samples were selected such that no additional verbal semantic cues to speaker identity were present. The famous voice set comprised 10 politicians, five actors, seven other media personalities from television or radio and two members of the British Royal family. The task on each trial was to name or provide other relevant biographical information about the speaker.

##### 2.3.3.3 Environmental sound identification

Identification of environmental sounds was assessed using a set of 30 common everyday sounds (listed in Supplementary Table S5 on-line), representing both mechanical (*n*=10) and natural (animal, human, water; *n*=20) sources. Sound samples were chosen to be frequently encountered, unique and clearly identifiable. The task on each trial was to name or provide other relevant semantic information, such as source description, about the sound; where sound identity was potentially non-unique (e.g., some mechanical sounds), half a point was given for an alternative potentially appropriate answer, for example, the sound of an engine as a washing machine.

##### 2.3.3.4 Face identification

In order to evaluate semantic processing of accents in relation to more general (non-auditory) processing of semantic information about people, identification of faces was assessed using photographs of the same 24 famous individuals used in the voice identification task described above (Hailstone et al., 2010; listed in Supplementary Table S4 on-line). Each face was presented as a black and white image on a notebook computer monitor. The task on each trial was to name or provide other relevant biographical information about the person whose face was shown.

### 2.4 Analysis of behavioural data

The performance of each patient on the experimental tests was compared with the healthy control group using analysis of variance tests adapted for small reference samples (after Crawford and Howell, 1998) under Stata^®^. A threshold *p*<0.05 was accepted as the criterion for a significant difference.

## 3 Results

### 3.1 General neuropsychological data

General neuropsychological data for all participants are summarised in Table 1. Relative to the healthy control group, both patients showed a profile of cognitive deficits in keeping with their clinical syndromes. Patient AA exhibited primary deficits in sentence comprehension and verbal repetition with spared single word comprehension and parietal function, in keeping with the diagnostic features characteristic of PNFA. Patient PA demonstrated a primary profile of profound naming difficulty underpinned by degraded single word comprehension and semantic knowledge compared to premorbid estimates and relative to the healthy control, group, consistent with a diagnosis of SD.

### 3.2 Experimental behavioural data

Experimental behavioural data for each control and both patients are summarised in Table 2.

#### 3.2.1 Apperceptive processing

On the perception of a change in accent task, AA showed a severe deficit relative to the healthy control group, whereas PA performed comparably to healthy control participants, In contrast, both AA and PA performed equivalently to controls on the phoneme change perception and speaker change perception tasks.

#### 3.2.2 Semantic processing

##### 3.2.2.1 Accent identification

On the accent identification task, healthy control participants performed equally well on identification of Australian, South African and Chinese accents and slightly better when identifying standard Southern English and American accents. AA showed a severe deficit of accent identification relative to the healthy control group, scoring near chance; PA showed a relatively mild though also statistically significant deficit. Both patients had particular difficulty in correctly identifying Chinese and South African accents.

##### 3.2.2.2 Voice identification

On the voice identification task, healthy control participants varied widely in their ability to name correctly the famous voices; however, their performance generally improved when supplying other biographical information to identify speakers. AA showed a marked deficit of voice identification: she was unable to name any of the voices presented and could supply additional biographical information for only three. PA showed an even more marked deficit of voice identification: she could name only one of the voices presented and was unable to provide any additional biographical information.

##### 3.2.2.3 Environmental sound identification

On the environmental sound identification task, healthy control participants made errors chiefly with identification of mechanical sounds. AA showed a severe deficit of environmental sound identification. She misidentified inherently more ambiguous mechanical sounds (camera timer, watch alarm, engine, shovel digging, metal file) as perceptually similar sound sources (‘signal’, door buzzer, washing machine, chopping wood, and ‘machine’, respectively). In addition AA tended to provide superordinate sound categories (e.g., she identified a crow caw as a bird sound) but also confused perceptually similar sounds (e.g., identifying a train horn as a musical instrument, car horns as ‘a band tuning up’ and seagulls as ‘children playing in a playground’). PA showed a less severe though still significant deficit of environmental sound identification. Of PA's errors, five were superordinate errors ((bird calls identified simply as ‘a bird’ and a lamb bleating as ‘an animal’), three were mechanical sounds misidentified as perceptually similar sound sources and one was a human sound (snoring) misidentified as an animal.

##### 3.2.2.4 Face identification

Face identification performance (like voice identification performance) varied widely within the healthy control group and consistently improved when participants supplied other biographical information to identify faces. AA showed impaired face naming but performed normally when supplying additional biographical information to identify faces. In contrast, PA showed a marked deficit of face identification: she could name only two of the faces presented and was only able to provide additional biographical information for a further two.

#### 3.2.3 Comparison of patients and healthy controls with lower educational attainment

In light of the discrepancy in mean years of education between the patients and the healthy control group, we examined patient performance specifically in relation to two healthy volunteers with fewer years of education (control participants *C*1 and *C*2 in Table 2, with 10 and 11 years of education, respectively). These control participants both performed comparably to the more highly educated remainder of the healthy control group on the experimental tests; and more particularly, AA and PA showed clear deficits on the experimental measures relative to both healthy participants with lower educational attainment.

## 4 Discussion

Here we have demonstrated distinct profiles of accent agnosia in two patients with progressive aphasia syndromes. AA showed markedly impaired perception of changes in accents despite intact perception of changes in phonemes and voices; in addition, she showed a severe deficit of accent identification. AA's preserved discrimination of other kinds of complex auditory information (embodied in phonemes and voices) suggests that her deficit of accent processing is not grounded in a more general deficit of auditory early perceptual coding: rather, this pattern suggests a primary and relatively specific deficit at the level of representation of accented syllables as auditory objects, i.e. an apperceptive agnosia for accents. By contrast, PA was able to perceive changes in accents, phonemes and voices normally, but showed a relatively mild deficit of accent identification; and while both patients showed deficits of voice and environmental sound identification, AA was able to identify (though not name) faces normally, whereas PA showed an additional deficit of face identification. The profile of deficits exhibited by PA suggests that she is able to represent accent characteristics accurately but is deficient in attributing meaning to those representations: i.e., PA has a primary associative agnosia for accents. The findings in PA further suggest that this defcit is in the context of a more general, multimodal semantic impairment. AA’s apperceptive deficit of accent processing is in line with previous evidence for apperceptive deficits of environmental sound processing and accent comprehension in patients with PNFA (Goll et al., 2010a; Hailstone et al., 2012). The contrasting multimodal semantic deficits exhibited by PA are in keeping with other evidence for impairment across semantic categories and modalities in SD (Bozeat et al., 2000; Goll et al., 2010a, 2010b; 2012; Hailstone et al., 2010; Luzzi et al., 2007; 2010; Piwnica-Worms et al., 2010; Josephs et al., 2008; Fletcher & Warren, 2011); and in particular, with previously described impairments of person knowledge associated with right temporal lobe atrophy (Bozeat et al., 2000; Hodges & Patterson, 2007; Thompson et al., 2004; Hailstone et al., 2010). We note the lower educational attainment of both patients versus the above-average mean overall years of education of the healthy control group, but consider this factor is unlikely to have materially influenced the findings in respect of the experimental tests of accent processing here. Educational attainment has not previously been shown substantially to influence performance on accent processing tasks among patients with dementias (Hailstone et al., 2012); moreover, the ‘excess’ educational attainment in our control group was concentrated mainly at university level, by which stage substantial exposure to accents (and relevant information about them) has already occurred through immersion in the dominant culture. In addition, AA and PA showed deficits of accent processing in comparison specifically to healthy control participants with lower educational attainment (Table 2), suggesting that any effect from educational attainment was not driving the results.

The profiles of impairment in these two cases provide evidence concerning the cognitive organisation of accent processing in relation to other kinds of complex sounds. While the selective impaiment of accent discrimination (versus other complex sounds) shown by AA suggests that she has an apperceptive agnosia for accent, the basis for her accompanying deficit of accent identification is less clear. This could, in principle, result from inaccurate accent representation at earlier processing stages or an additional, conjoint semantic deficit of accent processing. An additional semantic impairment in AA's case is favoured by the occurrence of associated deficits of voice and environmental sound identification and by her more marked deficit of accent identification compared with accent change perception. On the other hand, AA's ability to identify accents was more severely degraded than her ability to identify environmental sounds, and this disparity was further underlined by the different response procedures used in these two tests (forced choice for accent identification, free identification for environmental sounds). Taken together, this evidence argues that the profile of AA's accent agnosia may reflect an interaction between apperceptive and semantic mechanisms. Such an interaction would be in keeping with previous evidence concerning the processing of accents and other kinds of complex nonverbal sounds in neurodegenerative disease (Goll et al., 2010a, 2012; Hailstone et al., 2011,2012) and with theoretical models of the brain organisation of complex sound processing (Goll et al., 2010b); the perceptual confusions made by AA on the environmental sounds identification task here hints at a similar interaction in this other domain of complex sound processing. It is unclear whether an analogous interaction might also account for AA's profound associative phonagnosia, which occurred in the context of intact apperceptive processing of voice identity (Hailstone et al., 2010). Moreover we cannot exclude the possibility of a contributing, selective deficit at the level of early auditory perceptual encoding in AA’s case. This level of processing was not assessed here; however, early auditory perceptual deficits have been documented in the progressive nonfluent aphasias (Goll et al., 2010a,2011; Rohrer et al., 2012) and might potentially impact selectively on the processing of accent versus other auditory object-level attributes (Goll et al., 2010b).

Turning to the case of PA, the severity of her semantic impairment might be taken to vary for different kinds of auditory objects; however, while semantic deficits can be fractionated in SD, especially earlier in the course (Warrington, 1975), caution is needed in interpreting these apparent discrepancies in this case, due to the different response procedures and likely variation in intrinsic difficulty among the identification tests presented. The profile of PA's deficits, in particular her severe associative phonagnosia and prosopagnosia, are in keeping with current models of the hierarchical cognitive organisation of person knowlege (Bruce & Young, 1986; Burton, Bruce, & Johnston, 1990; Neuner & Schweinberger, 2000). PA's syndrome and the right anterior temporal lobe emphasis of the atophy profile in her case are further consistent with current neuroanatomical models of the neural representation of stored knowledge about familiar people (Hanley, 2011; Hailstone et al., 2011). However, it remains unclear to what extent accent attributes may also participate in the semantic representations of persons. Whereas accent transcends the identity of individual speakers, it may contribute importantly to speaker recognition in some cases (for example, speakers with highly salient or characteristic accents, an effect long exploited by actors and voice artists: Blumenfeld, 2002). We speculate that such a mechanism may have contributed to the severe impairment of voice identification exhibited by AA: accent here would be acting as a semantic association of speaker identity, and not (noting also that AA was able to distinguish speakers normally) simply in series with perceptual encoding of voices. However, we do not wish to over-emphasise this putative semantic interaction, which would likely be an idiosyncratic rather than a generic effect, and should be susbtantiated in future work.

The neuropsychological comparion of AA and PA with our previously reported cases of progressive associative phonagnosia (Hailston et al., 2010) is potentially of considerable theoretical interest. Of those earlier cases, patient QR exhibited severe impairments of voice identification and familiarity judgments with relatively preserved recognition of faces and environmental sounds, while patient KL exhibited severe impairments of both voice and face recognition, with relatively preserved recognition of musical instruments and environmental sounds. Both QR and KL demonstrated preserved ability to analyse perceptual properties of voices and to recognise vocal emotions. We are cautious in drawing comparisons between these and our present cases, as the patients vary not only in the anatomical and likely pathological substrates of their syndromes but were assessed using tests that are only partly convergent for the experimental measures of interest. In particular, accent processing was not assessed in the patients described previously (Hailstone et al., 2010). Broadly speaking, however, our previous case KL and present case PA had similar clinical syndromes; while our previous case QR had essentially normal environmental sound recognition, whereas our present case AA showed environmental sound agnosia, implying a potential dissociation between the respective cognitive mechanisms in these cases. It would therefore be reasonable to infer that cases PA and KL both fall within the SD syndromic spectrum, sharing a severe multimodal semantic impairment, whereas cases AA and QR represent somewhat more discrete syndromes of auditory semantic impairment associated with peri-Sylvian cortical atrophy. The essential point to draw from a consideraton of these cases together is that careful delineation of auditory apperceptive and semantic capacities for different categories of complex sounds in neurodegenerative syndromes reveals a fractionated, modular cognitive architecture that potentially parallels the neuropsychological modularity described previously in other sensory modalities (notably, vision) (Warrington, 1982; Goll et al., 2010b).

Clinical disorders of accent processing have seldom been described. This may simply reflect reporting bias (impaired processing of accents is much less likely to prove disabling than associated disorders of language or voice processing) and the challenges that studying such disorders entail. However, it may also reflect the availability of additional contextual cues (linguistic, vocal and extra-auditory) that tend to compensate for defective processing of accent in daily life. We argue that accent processing disorders have a disproprotionate theoretical importance since they potentially provide a rare window on apperceptive mechanisms of complex sound analysis and the representation of non-linguistic perceptual and semantic vocal characteristics. More pertinently, the patients described here provide further evidence for the breakdown of generic cortical auditory mechansims in PPA syndromes (Hailstone et al., 2010, 2011, 2012; Goll et al., 2010a,2010b,2011,2012; Rohrer et al., 2012), arguing for a reappraisal of such syndromes beyond language deficits. The present cases do not directly address the anatomical basis of accent processing deficits; however, the individual atrophy profiles in these cases (Fig. 1) are consistent with a previously demonstrated group-level anterior superior temporal lobe correlate of accent processing performance in Alzheimer's disease (Hailstone et al., 2012). These cases further suggest that profiles of impaired accent processing versus processing of other complex sounds may have some specificity for different PPA syndromes.

This study shares the limitations of all single case studies, requiring substantiation in larger patient cohorts with the opportunity for direct structural and functional neuroanatomical correlation. With specific reference to the organisation of accent processing, further work should examine earlier stages of auditory perceptual encoding with a more detailed analysis or manipulation of accent characteristics and probing a broader range of accents. The last is especially important in order to distinguish idiosyncratic deficits from more generic and informative profiles of impairment. From the perspective of cognitive neuroscience, there is considerable interest in establishing the extent to which accent processing interacts with (or may dissociate from) the processing of other vocal and nonvocal complex sound attributes, since this will act as a test case for assessing the valdity of current models of cortical auditory processing (Goll et al., 2010b). Particularly relevant in this regard might be a comparison of the processing of accents with prosody, another form of suprasegmental vocal pattern that is put to quite distinct behavioural use in coding emotional and lingusitic intonations. From a clinical neurobiological perspective, accent processing offers a novel model paradigm with which to probe brain network disintegration underpinning focal neurodegenerative diseases.

## Figures and Tables

**Table 1:** Summary of general demographic and cognitive data for all participants.

**Characteristics**	**AA**	**PA**	**Healthy controls**⁎ *n*=10^†^
***General***			
Age (years)	67	71	66 (57–71)
Education (years)	11	10	16 (10–20)
Symptom duration (years)	2	3	N/A
MMSE (max 30)	26	28	N/A
Verbal IQ	**78**	**84**	121 (106–130)
Performance IQ	97	93	120 (88–141)
***Language***			
BPVS (max 150)	**126**	**136**	147 (139–150)
GNT (max 30)	**0**	**3**	26 (19–29)
NART (max 50)	**12**	**27**	44 (30–49)
***Arithmetical and spatial***			
GDA addition (max 12)	5	5	6.9 (4–11)
GDA subtraction (max 12)	**4**	6	8.7 (6–12)
VOSP (max 20)	19	18	17 (13–20)
***Executive***			
Stroop: Colour naming (time in seconds)	48	27	28 (24–36)
Stroop: inhibition (time in seconds)	72	60	52 (36–70)
Digit span reverse (maximum string length)	5	6	5 (4–7)

Key: ^⁎^mean (range) data shown. Patient data below healthy control range are shown in bold. ^†^two healthy control participants did not complete general neuropsychological assessments; BPVS, British Picture Vocabulary Scale (McCarthy & Warrington, 1992; Lloyd et al., 1982); GNT graded naming test; GDA, Graded Difficulty Arithmetic (Jackson & Warrington, 1986); IQ, scores calculated from the Wechsler Abbreviated Scale of Intelligence (Wechsler, 1999); MMSE, Mini-Mental State Examination score; NART, National Adult Reading Test; Stroop, D-KEFS Stroop test (Delis, Kaplan, & Kramer, 2001); VOSP, Visual Object and Spatial Perception battery.

**Table 2:** Summary of experimental behavioural data for all participants.

**Experimental tests**	**AA**	**PA**	**Healthy controls**												
			Group mean	*C*1^†^	*C*2^†^	*C*3	*C*4	*C*5	*C*6	*C*7	*C*8	*C*9	*C*10	*C*11	*C*12
***Apperceptive***															
Phoneme discrimination (/36)	94	94	97	100	100	100	100	94	89	100	94	89	97	100	100
Speaker change (/28)	93	93	93	89	93	100	96	89	96	89	96	82	86	100	75
Accent change (/30)	**60**^⁎⁎⁎^	86	90	93	90	90	90	87	83	93	80	93	93	97	90
***Semantic***															
Famous person (/30)															
*Faces: naming*	**33**^⁎⁎^	**8**^⁎⁎^	82	83	71	97	92	54	79	100	63	85	100	83	63
*Faces: all bio*	96	**17**^⁎⁎⁎^	98	100	92	100	96	96	94	100	100	96	100	100	96
*Voices: naming* (*/*)	**0**^⁎^	**4**^⁎^	71	50	50	96	88	69	65	100	42	77	75	79	83
*Voices: all bio*	**13**^⁎⁎^	**4**^⁎⁎^	82	58	58	100	88	96	81	100	67	85	75	100	92
Environ sounds identification (/30)	**67**^⁎⁎⁎^	**77**^⁎⁎^	93	93	93	97	93	87	90	97	87	97	93	97	87
Accent Identification (/30)	**23**^⁎⁎⁎^	**67**^⁎⁎^	88	77	87	87	93	90	93	90	87	87	93	83	80

*Standard Southern English* (*/*6)	5	6	5.8	6	6	5	6	6	6	6	6	5	6	6	5
*American* (*/*6)	2	6	5.6	6	6	6	6	6	6	5	6	6	5	4	5
*Australian* (*/*6)	1	5	4.9	3	5	5	6	5	5	6	5	6	4	3	6
*Chinese* (*/*6)	0	2	5.1	6	6	5	4	5	6	3	6	4	5	5	6
*South African* (*/*6)	0	1	4.8	5	5	6	6	5	3	5	5	4	4	6	4

All scores have been normalised to percentage scores correct. Patient scores below the healthy control range are shown in bold. Key: ^⁎^*p*<0.01, ^⁎⁎^*p*<0.001, ^⁎⁎⁎^*p*<0.0001 (based on Crawford and Howell, 1998); ^†^healthy individuals with lower educational attainment; bio, biographical information (percentage identified correctly by any biographical information, including naming); *C*, healthy control participant.

## Footnotes

Appendix A Supplementary data associated with this article can be found in the online version at doi:10.1016/j.neuropsychologia.2013.05.013.
