# Supplementary material for: Agnosia for accents in primary progressive aphasia
Source: Neuropsychologia. 2013 Aug;51(9):1709–15. doi: 10.1016/j.neuropsychologia.2013.05.013 (PMC3724054; doi:10.1016/j.neuropsychologia.2013.05.013)
Supplement: Supplementary file 1 — Supplementary Material [file mmc1.docx]

**SUPPLEMENTARY MATERIAL for**

**Agnosia for accents in primary progressive aphasia,** by PD Fletcher et al

**Table S1. Words used to construct sequences in the accent discrimination test**

Abstain

American

Bark

Birch

Chair

Child

Delve

Democracy

Denude

Erratic

Essay

Festoon

Finery

Fixation

Flask

Glimmer

Ground

Guild

Isle

Liaison

Limp

Lubricate

Mole

Munch

Muscle

Mutinous

Neutral

Nicotine

Noose

Notify

Notion

Porcupine

Prosper

Protector

Quibble

Quirk

Revulsion

Ridicule

Rinse

Skeleton

Skimp

Slang

Stimulant

Strike

**Table S2. Words presented in the phoneme pairs discrimination test**

| *Same* | Leaf | *Different* | Pill | Bill |
| --- | --- | --- | --- | --- |
|  | Write |  | Tap | Pat |
|  | Deed |  | Bone | Moan |
|  | Fall |  | Bomb | Mob |
|  | Tape |  | Moan | Gnome |
|  | Nip |  | Seed | Feed |
|  | Pail |  | Veil | Fail |
|  | Dote |  | Don | Nod |
|  | Gut |  | Live | Life |
|  | Down |  | Dab | Bad |
|  | Pig |  | Hen | Head |
|  | Sack |  | Cut | Cup |
|  | Bat |  | Neat | Meat |
|  | Rung |  | Nail | Lane |
|  | Dale |  | Cut | Tuck |
|  | Coat |  | Lean | Kneel |
|  | Fang |  | Mane | Name |
|  | Road |  | Ted | Debt |

**Table S3. Sentences used in the accent identification test**

Sarah Perry was a veterinary nurse who had been working daily at an old zoo in a deserted district of the territory

That area was much nearer to her and more to her liking

So she was very happy to start a new job at a superb private practice in North Square, near the Duke Street tower

Even so, on her first morning she felt stressed

She ate a bowl of porridge, checked herself in the mirror, and washed her face in a hurry

Then she put on a plain yellow dress and her fleece jacket, picked up her kit and headed for work

**Table S4. Famous people in the face and voice identification tests**

David Attenborough

Alan Bennett

Tony Blair

Gordon Brown

George Bush

Charles Prince of Wales

Bill Clinton

Billy Connolly

Ronnie Corbett

Judy Dench

Diana Princess of Wales

Bob Geldof

Edward Heath

John Humphries

Neil Kinnock

Joanna Lumley

John Major

Ian Paisley

Jonathan Ross

Janet St Porter

Margaret Thatcher

Anne Widdecombe

Kenneth Williams

Terry Wogan

**Table S5. Environmental sounds in the sound identification test**

ambulance siren

man belching

camera timer

car horns

chicks cheeping

cockerel

cow

crow

dog barking

car engine

horse neighing

horse hooves

lamb bleating

woman laughing

mosquito

pigeon cooing

pouring liquid from a bottle

metal saw

seagulls

shovel in soil

baby sneezing

man snoring

stream

surf

telephone ringing

telephone receiver being replaced

train horn

train whistle

watch alarm

waterfall
